# Social Visual Perception Under the Eye of Bayesian Theories in Autism Spectrum Disorder Using Advanced Modeling of Spatial and Temporal Parameters

**DOI:** 10.3389/fpsyt.2020.585149

**Published:** 2020-09-23

**Authors:** Chara Ioannou, Divya Seernani, Maria Elena Stefanou, Monica Biscaldi-Schaefer, Ludger Tebartz Van Elst, Christian Fleischhaker, Giuseppe Boccignone, Christoph Klein

**Affiliations:** ^1^ Department of Child and Adolescent Psychiatry, Psychotherapy and Psychosomatics, Medical Faculty, University of Freiburg, Freiburg, Germany; ^2^ School of Psychology and Clinical Language Sciences, University of Reading, Reading, United Kingdom; ^3^ Department of Psychiatry and Psychotherapy, Medical Faculty, University of Freiburg, Freiburg, Germany; ^4^ Department of Computer Science, University of Milan, Milan, Italy; ^5^ Department of Child and Adoelscent Psychiatry, University Hospital Cologne, Cologne, North Rhine-Westphalia, Germany; ^6^ Department of Psychiatry, School of Health Sciences, National and Kapodistrian University of Athens, Athens, Greece

**Keywords:** Bayesian theories, social perception, eye movements (EM), hidden Markov models (HMM), advanced analysis, autism spectrum disorder (ASD)

## Abstract

Social interaction in individuals with Autism Spectrum Disorder (ASD) is characterized by qualitative impairments that highly impact quality of life. Bayesian theories in ASD frame an understanding of underlying mechanisms suggesting atypicalities in the evaluation of probabilistic links within the perceptual environment of the affected individual. To address these theories, the present study explores the applicability of an innovative Bayesian framework on social visual perception in ASD and demonstrates the use of gaze transitions between different parts of social scenes. We applied advanced analyses with Bayesian Hidden Markov Modeling (BHMM) to track gaze movements while presenting real-life scenes to typically developing (TD) children and adolescents (*N* = 25) and participants with ASD and Attention-Deficit/Hyperactivity Disorder (ASD+ADHD, *N* = 15) and ASD without comorbidity (ASD, *N* = 12). Regions of interest (ROIs) were generated by BHMM based both on spatial and temporal gaze behavior. Social visual perception was compared between groups using transition and fixation variables for social (faces, bodies) and non-social ROIs. Transition variables between faces, namely gaze transitions between faces and likelihood of linking faces, were reduced in the ASD+ADHD compared to TD participants. Fixation count to faces was also reduced in this group. The ASD group showed similar performance to TD in the studied variables. There was no difference between groups for non-social ROIs. Our study provides an innovative, interpretable example of applying Bayesian theories of social visual perception in ASD. BHMM analyses and gaze transitions have the potential to reveal fundamental social perception components in ASD, contributing thus to amelioration of social-skill interventions.

## Introduction

Autism Spectrum Disorder is a heterogeneous disorder, with qualitative impairments in social interaction being one of the cardinal symptoms (1). Considering the challenges these symptoms evoke in everyday life of affected people and the need for effective interventions, many theories in autism spectrum disorder have laid their focus upon social cognition (2–4). Some of the most recent and promising theories are the Bayesian theories of autism spectrum disorder, which also unify a wide range of its clinical characteristics. In this Bayesian framework, it is suggested that perception in autism spectrum disorder is atypically resistant to prior acquired information, thus the affected individual engages with an “almost always new” input in probabilistic terms when experiencing an event (5). This implicit divergence in predictive coding is also referred to as «Hypothesis of Predictive Impairment in Autism» (6). Regarding social perception, these theories can explain manifestations of autism spectrum disorder such as atypicalities in apprehending social stimuli, inferring social causalities, predicting social intention or developing a regulating system for social processes (7).

Social interactions can be more demanding, complicated and unpredictable than one tends to believe (8). A basic source of social perception is visual and thus the orientation of gaze is crucial for the observer in order to comprehend social signals (9). Therefore, recording of gaze movements with the eye-tracking technique represents an objective measure in studying social perception (10). Common practices include measurements of fixation direction and duration at different parts of social scenes. Usually, stimuli are differentiated in social (e.g. faces, bodies) and non-social elements (e.g. objects) (11). Such paradigms have been widely investigated and particularly eye tracking provides insights about the underlying mechanisms of the social understanding in autism spectrum disorder (12, 13). On the one hand some studies report that participants with autism spectrum disorder prefer looking to non-social in comparison to social stimuli (14) and less to faces compared to non-face parts of a social scene (15). Moreover, regarding atypicalities in following the gazes of others, deficits in joint attention have been shown in participants with autism spectrum disorder (16). On the other hand, there are studies reporting no difference regarding social attention between participants with autism spectrum disorder and their typically developed peers (17). Also, it has been reported that deficits of joint attention are specifically found in its initiation part and not in the response to joint attention (18). Albeit such inconsistencies, studies suggest that participants with autism spectrum disorder focus less on social elements than typically developing controls (19).

However, with the established methods of gaze analysis, the actual process of social perception is limited to a quantification of attention isolated to specific areas. Particularly, they mostly refer to fixation variables, in terms of time spent in predefined, manually drawn, regions of the scene (20). This quantification of social focus neglects the importance of actively combining different aspects of the stimuli, for example the gaze connection between two interacting people in a social scene. Nevertheless, the linking between informational areas has been reported to reflect the actual gaze patterns of individuals (21). In gaze movement terms, the linking of visual inputs is defined as transitions and refers to the saccadic paths between Regions of interest (ROIs), namely a cluster of fixations gathered in an informative area, e.g. a face or a body. Moreover, in order to perceive social information, there is a tendency to orient the gaze to the face, particularly the eyes of the presented person (22). Therefore, when looking at a scene of people interacting, it is likely to orient the gaze from one face to another, as part of social content exploration (23). Consequently, linkage of socially relevant areas is neither completely random nor completely deterministic and is thus related to a certain degree of probability (24).

Leaning on Bayesian theories of autism, social understanding in autism spectrum disorder can also be atypical at the level of engaging with the aforementioned probabilistic associations, namely gaze transitions. Prior inputs and the inference of further outputs are regulated by the strength of these associations (25) which in visual perception can be manifested in the interdependence of successive fixations. In other words, the current point of gaze is influenced by the previous one and further the current step affects the next gaze shift (26). Moreover, the actual attentional processing of the individual looking at a scene remains “hidden” in the sense that it cannot be observed directly but can be inferred through the gaze movements. Under this perspective, the underlying mechanisms of the atypical social perception in individuals with autism spectrum disorder are still unclear and require analyses that treat social visual perception as a process that unfolds in space and time and accounts for its probabilistic components.

Our study applies Bayesian Hidden Markov Modeling (BHMM) (21, 27, 28) to capture both dynamics and structure of visual exploration of social scenes in autism spectrum disorder and typical developed (TD) children and adolescents. A BHMM represents the moment-to-moment evolution of a random variable whose values are observable (the observable stochastic process) and generated conditionally on the temporal evolution of a hidden random variable (the hidden or latent process). The hidden variable can take values over a finite number of states and the state at the current timepoint only depends on the state at the previous timepoint, representing a Markovian process. In the example of eye tracking, gaze data can be addressed as time-series of a stochastic Markovian process (29). Both the model selection (number of ROIs) and the learning of the BHMM parameters are completely data driven. Further, the specific kind of BHMM we are exploiting here (30) also allows for the hierarchical representation and inference of group gaze behavior and attention dynamics (see also Methods section 2.4.1). We surmise that prior distributions on individual and group parameters of gaze behavior inferred at the data analysis stage can be usefully integrated with Bayesian models of sensory input in order to provide a broader sensorimotor account of atypical behavior in ASD.

Finally, the recent DSM-V diagnostical guidelines (1) recognize the comorbidity of autism spectrum disorder with other conditions and enable for the first time their simultaneous diagnoses. Particularly it is reported that around 70% of individuals with autism spectrum disorder might also fulfill the criteria for another co-morbid mental disorder and approximately 40% can have two or more co-morbid mental disorders (1). Common comorbid conditions include anxiety disorders, epilepsy, depression, gastrointestinal symptoms, challenging behavior, sleep problems, toileting, and feeding issues (31–36), as well as Attention-Deficit/Hyperactivity Disorder (ADHD) (37). Interestingly, even before DSM-V, studies have reported ADHD traits in populations with autism spectrum disorder in the areas of attention and social cognition (38). Moreover, symptoms of ADHD in autism spectrum disorders have been also associated with impairments in executive function (39). In our study, considering the feasibility of a dual diagnosis of ASD and ADHD, we included autism spectrum disorder participants both with comorbid ADHD (from now on referred to as ASD+ADHD) and without (from now on referred to as ASD). Thus, we consider current diagnostic recommendations and the emerging research in the comorbid group, highlighting its clinical significance and manifestation (40, 41).

Thus, our study aims to demonstrate a novel approach in psychiatric research applying BHMM to gaze data of ASD, ASD+ADHD participants and TD controls with stimuli depicting real-life social scenes. Apart from considering the comorbid group as a separate entity, other novelties of our approach from a methodological standpoint include: i) the feasibility of applying advanced analytical methods to clinical populations, ii) the investigation of both fixation and interpretable transition variables and, more subtle, iii) the integration of both i) and ii) in the principled framework of Bayesian inference. Beyond the technical novelties that to the best of our knowledge have not been exploited in this research field, this latter aspect paves the way to bridge advanced methods for data analysis with Bayesian theories of autism spectrum disorder. In particular, approaches focusing on active inference (42) suggest that the core aspects of autism relate fundamentally to how individuals sample the world. Indeed, the very idea of sampling is at the heart of the BHMM: giving the current state, sampling the next ROI and, in turn, sampling the new gaze position. We hypothesize that compared to the control group, the autism spectrum disorder groups will show less dynamical links, in terms of gaze transitions, between the socially relevant ROIs and also fixate less the presented social elements.

## Methods

### Participants

Inclusion criteria were normal or corrected-to-normal vision, native-speaker level in the local language, age between 10 and 14 years and intelligence quotient (IQ) within two standard deviations at the lower end (≥70) (43). Exclusion criteria were strabismus, diagnoses of Tourette syndrome, specific reading disorder, epilepsy or other neurologic disorders, severe psychiatric disorders like schizophrenia, acute depressive episode, suicidal ideation and any known ASD- or ADHD-related specific genetic syndrome. Initially, 59 participants were examined (26 TD, 18 ASD+ADHD and 15 ASD). Recordings of seven participants not fulfilling eye-tracking quality criteria (see below in *Apparatus*) were excluded (1 TD, 3 ASD+ADHD and 3 ASD). Finally, data of *N* = 52 participants were available for analysis, namely *N* = 25 TD (average age: ± one standard deviation 12.1 ± 1.5 years; 52% male; IQ: 110 ± 17), *N* = 15 participants with ASD and co-morbid ADHD (ASD+ADHD; age: 12.0 ± 1.0 years; 93% male; IQ: 96 ± 15) and *N*= 12 participants without comorbidity (ASD; age: 12.3 ± 1.1 years; 83% male; IQ: 103 ± 21). Diagnoses were made by a group of clinical psychologists and child and adolescent psychiatrists based on International Classification of Diseases (ICD-10) criteria. Details on diagnostic tests and screenings applied are described in the **Supplementary Data**. Other comorbidities included for the ASD group: diurnal/nocturnal enuresis (*N* = 2), specific insect phobia (*N* = 1), developmental dyspraxia (*N* = 1), mixed receptive-expressive language disorder (*N* = 1), chronic tic disorder (*N* = 1), obsessive compulsive disorder (*N* = 1); and for the ASD+ADHD group: diurnal/nocturnal enuresis (*N* = 1), adjustment disorder (*N* = 1), vocal tic disorder (*N* = 1), expressive language disorder (*N* = 1). The study was approved by the institutional ethics committee. All participants and their parents gave their written informed consent before participation.

### Apparatus

Participants were seated in a sound attenuated cabin, with a distance of 70 cm from a 24”-screen (1920 × 1080 pixels, 60 Hz). Stimuli were presented with Presentation^®^ software (version 17.2, Neurobehavioral Systems, Inc., Berkley, CA). Binocularly gaze recordings were accomplished with the iView RED250 system (Senso-Motoric Instruments GmbH, SMI, Teltow, Germany; sampling rate: 120 Hz). Based on 5-point calibration an accuracy of <0.05° was achieved for every participant. Data were preprocessed and exported with SMI-BeGaze version 3.7, using its default fixation classification, where fixations are defined as events of minimum 60 milliseconds and with a maximum dispersion of 2 degrees. Blinks and off-screen fixations were excluded. To ensure adequate gaze data quality only data sets with a tracking ratio >75% and a total fixation time above 50% of the stimulus duration had been included.

### Stimuli and Procedure

The participants completed a battery of eye-tracking tasks, totaling 90 minutes with breaks, while for the free-viewing stimuli, two pairs of stimuli with real-world pictures were presented – two depicting one actor and two depicting four actors. Total task duration, including instruction and calibration, was less than 10 minutes and eye movements were recorded for 120 seconds per stimulus. Beforehand, questioning about the pictures was announced to ascertain the attention of the participant. An experimenter sat with the participant in the cabin and care was taken to adapt task progression to each participant’s demands.

### Data Analysis

#### Markov Modeling

In the present study, we use a Bayesian Hidden Markov Model (BHMM) as previously described (27, 28). The BHMM relies on a ﬁnite number of hidden states (in this study, Regions of Interest, ROIs) and contains a vector of prior values, which indicates the probability of a time-series beginning with each state; a transition matrix, which parameterizes the transition probabilities between any two hidden states; and a Gaussian emission for each state, which represents the probabilistic association between recorded eye ﬁxation locations and a hidden state. Different from a classic, frequentistic Hidden Markov Model (HMM), a Bayesian HMM treats the HMM parameters (e.g., the transition matrix) as random variables and assigns Gaussian emissions on such parameters. At the learning stage, a Bayesian variant of the parameter estimation algorithm automatically penalizes model complexity *via* the distributions on the model parameters. A variational Bayesian expectation maximization (VBEM) algorithm is being used starting from the priors and learning the model parameters in a data-driven way. Further, it provides the option for learning shared fixation patterns and producing representative ROIs, by clustering individual BHMMs using a variational hierarchical expectation maximization (VHEM) algorithm (30).

The *scanpath modeling and classification with hidden Markov models* toolbox (27, 28) was applied in MATLAB (R2018b, The MathWorks, Natick, MA, United States) for the analysis following, separately for the picture with four depicted actors (stimulus A) and one depicted actor (stimulus B). Default hyperparameters were used.

**Figure 1:**
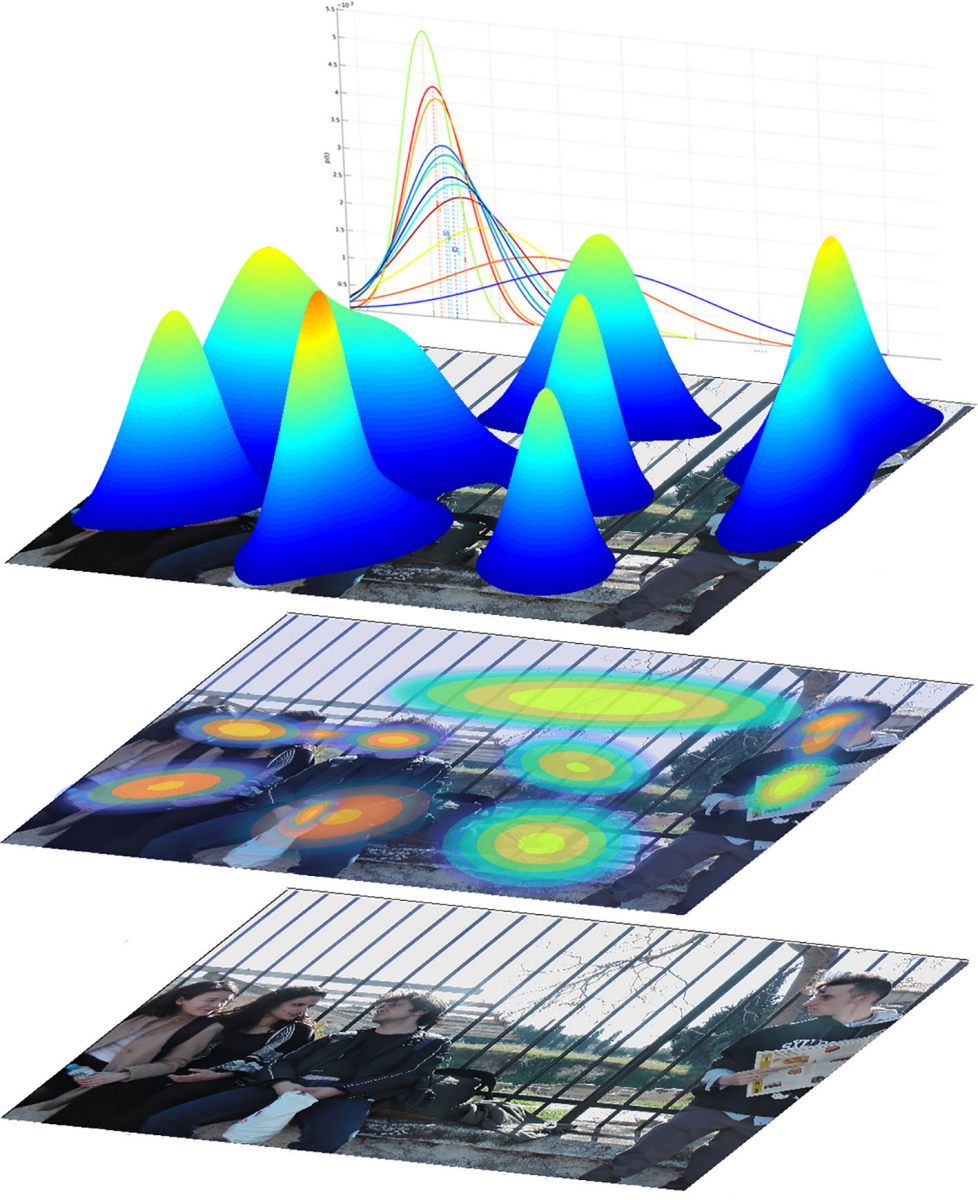
Illustration of the composition of Regions of Interest (ROI) and the process of their generation. The ROIs depicted here were formed from gaze data of all participants, including the fixation durations. i) lower layer: the input stimulus A. ii) middle layer: the stimulus with separate modeled ROIs, depicted as flattened two-dimensional Gaussian distributions, where the concentric ellipses depict the probability of a fixation belonging to that ROI, decreasing from center to periphery. iii) upper level: an alternate illustration of the same Gaussian distributions, here in three-dimensional representation. In the background the Gaussian distributions of the fixation duration are depicted, which for each ROI were also based on the data of all participants.

In this study each participant was summarized by an individual HMM, the observable process being the gaze positions that the participant generates while sequentially deploying attention to a finite number of ROIs. The process analysis was composed of the following steps:

Step 1) Scan-paths of all participants from every group are clustered by BHMMs with the goal of generating representative ROIs (see **Figure 1**).

Step 2) Scan-paths of each participant are modeled by individual BHMMs, which are initiated from the representative ROIs found in step 1.

Two features describe the data-driven generation of attentional states for both steps. Firstly, the ROIs are assumed to be the hidden attentional states. While the participant is dwelling on a specific ROI, a number of actual gaze positions is generated by sampling from an emission probability distribution, with a decreasing probability from center to periphery. Thus, a single fixation receives a posterior probability of belonging to a ROI and is assigned to the ROI where it exhibits the highest probability. Secondly, during the process of ROI generation the above modeling additionally considers a temporal third dimension, namely the duration of every single fixation. Thus, the term “regions” of interest in this study, although potentially misleading at the first glance, does not refer to a spatial entity alone, but also incorporates the temporal component of each fixation duration as well as its displacement in the x and y axis. Importantly, this temporal component considers the processing speed in terms of fixation duration of every single fixation (44). Moreover, the temporal dimension incorporated in the BHMM accounts for the processing of visual attention as it unfolds in time (27).

#### Definition of Gaze Variables

The ROIs were categorized in *social* and *non-social*, former consisting of depicted faces and bodies and latter included ROIs containing objects and background. For the purpose of this study we define transition and fixation variables as follows.

Transition variables investigate the dynamical links between any combination of ROI pairs and can be expressed as *transition count* and *transition probabilities*. Transition count is the actual amount of unidirectional transitions between two given ROIs. The Bayesian transition probability is an essential part of the BHMM and expresses for each individual the probability of a unidirectional transition taking place between two given ROIs during the experiment. For our analysis, we considered social-to-social and non-social to non-social ROI pairs (hereinafter referred as social and non-social transitions). The social transitions were composed of the following transition pairs: face to face, body to body, face to body and body to face.

Additionally, established fixation variables were calculated based on the three BHMM ROI types (faces, bodies and non-social ROIs). *Fixation* and *visit count* were defined as the number of fixations or visits in a specific ROI category, respectively. A visit describes the event, beginning from the entrance of the gaze into a ROI and ending at its exit. Thus, a visit can contain one or more fixations (45).

Finally, *total fixation duration*, defined as the total amount of time spent on a stimulus, regardless of ROI, and *total transition count* representing the total number of transitions performed in one stimulus were calculated.

### Statistical Analysis

Linear mixed effects models were applied separately for each gaze variable and each ROI category similar to (46) using the statistical software R (R Core Team, v 3.6.1) and the lme4 library (v 1.1-19). In general, the patient group was the main effect term of interest. The number of depicted actors and the interaction term of the two main effects served as additional fixed effects, although not of primary interest. Before introducing total fixation duration or total transition count as covariates in the model, the effect of patient group on these variables was examined (47). P-value <0.05 was used for statistical significance.

For the transition variables the model was structured as follows:

transition variable ∼1+ group + actors +group: actors + total transitions + (1 | participant)

where participant group (TD, ASD+ADHD, ASD) and number of depicted actors (one or four) were the fixed effects with an interaction term. The covariate total transitions per stimulus was centered at its mean. The analysis was performed for the five available ROI combinations and analogous model structures were used for the variable of transition probabilities. Due to the nature of the later variable no covariate term was applied in this case.

For the basic fixation variables (fixation and visit count) the model was structured with the same fixed effect terms as above and with individual mean-centered total fixation duration (in seconds) per stimulus as a covariate, separately for the three ROI types:

fixation variable ∼ 1 + group +actors + group: actors + total fixation duration + (1 | participant)

## Results

### Generated ROIs

**Figure 2:**
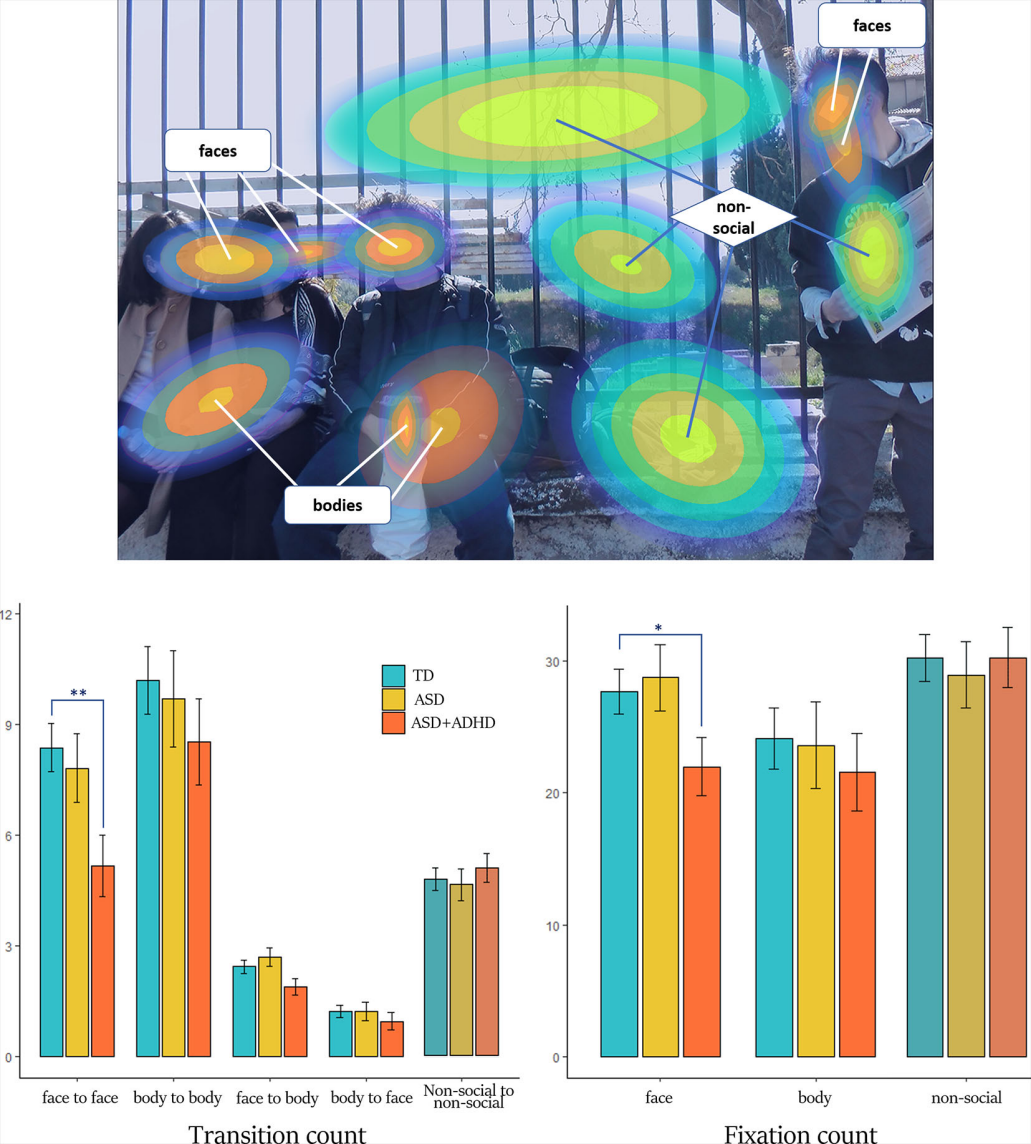
Generated ROIs and results for transition and fixation count. Upper: The ROIs of stimulus A formed from the gaze data of all participants are shown as Gaussian distributions, categorized in “social” (composed by faces and bodies) and “non-social”. The probability of a fixation belonging to a ROI is decreasing from center to periphery. Here, the outer borders of the ROIs represent the 95% of the Gaussian distribution. Lower left: transition count for the different types of transition and groups. Lower right: fixation count for the different ROI categories and groups. Bar charts are composed of mean estimates and standard error. Color-matching to groups is explained in the figure legend. Note the statistic significant lower the transition and fixation count for the ASD+ADHD group compared to the TD for the ROI of faces (* denotes p-value < 0.05, ** denotes p-value < 0.01).

The process of ROI generation by the BHMM is visualized in **Figure 1**, exemplary for stimulus A. The ROIs represent Gaussian distributions of probabilities at x-y coordinates, which can be best understood as 3D Gaussian bells with the highest probability in the center and decreasing probability to the periphery. In order to semantically separate the elements of the scenes, the optimal number of ROIs was found to be *N* = 10 for stimulus A and *N* = 13 for stimulus B. Additionally, the temporal dimension of each single fixation duration is integrated. Thus, the gaze variables investigated here, based on the BHMM and the respective ROIs, represent the processing of visual attention evolving in time and consider the temporal aspect of attention processing. Considering the categorization into social and non-social ROIs, one BHMM state of each stimulus representing sparse fixations with the longest durations and no regional pattern was not included, resulting in 12 ROIs for stimulus A and 9 ROIs for stimulus B. For stimulus A 8 ROIs were categorized as social (5 for faces and 3 for bodies), while for stimulus B there were 2 social ROIs (1 face and 1 body) (see also **Figure 2** and **Supplementary Figure S1**).

### Transitions

The total count of transitions for stimulus A was on average ± one standard deviation 304 ± 56, 290 ± 57, and 302 ± 66 for groups TD, ASD+ADHD and ASD, respectively. For stimulus B it was 298 ± 52, 293 ± 49, and 300 ± 32, respectively. The total transition count did not differ statistically neither between groups nor stimuli (*p* = 0.808 and 0.843, respectively, see **supplementary Table S1**). It was nevertheless added as a covariate to the subsequent analysis, for reasons of completeness (47) (see **Supplementary Table S1**).

#### Social Transitions

**Figure 3:**
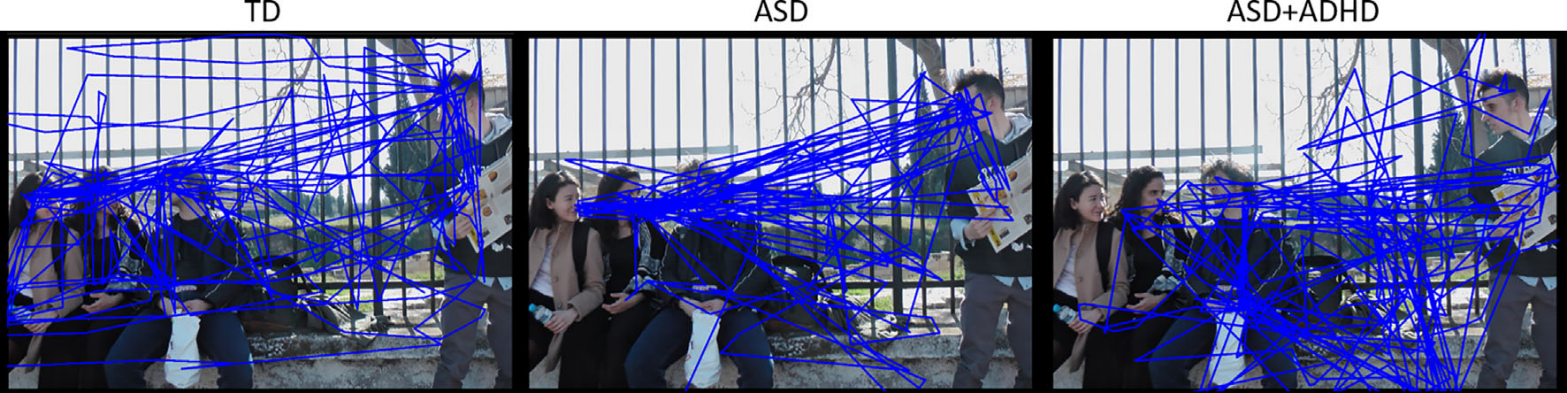
Gaze trajectory recordings of three different exemplary participants (from left to right: TD, ASD and ASD+ADHD). Note the differences in exploration, transitions between faces and non-social ROIs.

The total count of social transitions was on average 96 ± 26 for the TD group, 83 ± 23 for the ASD+ADHD group and 96 ± 21 for the ASD group (see also **Supplementary Table S2**). Mixed-effects analysis revealed a significant effect of clinical group, regarding transitions between faces. Compared to the TD group, transition count between faces was overall lower for the ASD+ADHD group (mean estimates ± standard error 5.2 ± 0.8 vs 8.4 ± 0.6, *p* = 0.002, see **Table 1**), while the ASD group did not differ (7.8 ± 0.9). **Figure 3** illustrates gaze trajectory recordings for one exemplary participant out of each group. Noticeably, the participant of the comorbid group avoided transitions between faces, whereas the ASD participant showed a rigid pattern in comparison to the TD participant.

**Table 1:** Results of mixed effects analyses on transition counts and probabilities for social and non-social ROIs.

	F-value	Estimated means			p-value	
	*Group Effect*	*TD*	*ASD*	*ASD + ADHD*	*TD vs. ASD*	*TD vs. ASD+ADHD*
**A. Transition count**						
face to face	4.86	8.4 ± 0.6	7.8 ± 0.9	5.2 ± 0.8	0.622	0.002**
body to body	0.62	10.2 ± 0.9	9.7 ± 1.3	8.5 ± 1.2	0.757	0.267
face to body	3.1	2.4 ± 0.2	2.7 ± 0.3	1.9 ± 0.2	0.406	0.057
body to face	0.5	1.2 ± 0.2	1.2 ± 0.3	1.0 ± 0.2	0.972	0.357
non-social ROIs	0.3	4.8 ± 0.3	4.6 ± 0.4	5.1 ± 0.4	0.757	0.549
**B. Transition probability**						
face to face	3.2	30 ± 2	26 ± 3	22 ± 2	0.251	0.012*
body to body	0.4	38 ± 2	39 ± 3	36 ± 3	0.721	0.540
face to body	1.4	11 ± 1	11 ± 1	13 ± 1	0.730	0.098
body to face	0.1	9 ± 1	8 ± 1	8 ± 1	0.915	0.618
non-social ROIs	0.4	24 ± 1	23 ± 1	24 ± 1	0.439	0.980

F-value for the fixed effect of clinical group and post-hoc tests for groups (with p-value) are listed. Estimated mean values are mean ± standard error. Mean estimate values for transition probabilities are in %. * denotes p-value < 0.05, ** denotes p-value < 0.01.

ASD, Autism Spectrum Disorder; ADHD, Attention Deficit and Hyperactivity Disorder; ROIs, Regions of interest.

The other social transitions were lower in the ASD+ADHD compared to the TD group, nevertheless did not differ statistically. Particularly, the transition count from body to body and 8.5 ± 1.2 vs 10.2 ± 0.9 (*p = 0.267*), face to body: 1.9 ± 0.2 vs 2.4 ± 0.2 (*p =* 0.057) and body to face: 1.0 ± 0.2 vs 1.2 ± 0.2 (*p =* 0.357). The ASD group did not show any statistically significant different transition values to the TD group for any of the four social categories (see **Table 1**).

Similar results were shown for transition probabilities, where transition between faces was significantly different, with lower values for the ASD+ADHD group (mean estimates ± standard error, 22 ± 2%) compared to the TD group (30 ± 2%, *p* = 0.012), while there was no difference for the other transition categories or for the ASD group. The complete results are summarized in **Supplementary Tables S3, S4**.

#### Non-Social Transitions

The total count of non-social transitions was on average ± one standard deviation 146 ± 41 for the TD group, 150 ± 44 for the ASD+ADHD group and 148 ± 40 for the ASD group. The ASD+ADHD group showed higher, yet not-significantly different, transition counts in non-social ROIs (average ± standard error: for TD 4.8 ± 0.3, for ASD+ADHD 4.6 ± 0.4, for ASD 5.1 ± 0.4) and similar transition probabilities (average± standard error: for TD 0.24 ± 0.01, for ASD+ADHD 0.24 ± 0.01, for ASD 0.23 ± 0.01, non-significant, see **Supplementary Materials S3, S4**).

### Fixation Variables

The variable of total fixation duration, used as a covariate for the subsequent analyses, did not differ statistically between groups or between stimuli (*p-value=0.158 and 0.549*, respectively, **Supplementary Table S1**). Compared to the TD group, the number of fixations on faces was lower in the ASD+ADHD group (average ± standard error: 22 ± 2.2 vs 27.7±1.7, *p =* 0.046), while there were no significant differences to the ASD group (28.7 ± 2.5) (see **Figure 2** and **Supplementary Table 5**). The number of visits to faces did not significantly differ between groups (average ± standard error: for TD 16.1 ± 1.0, for ASD+ADHD 14.8 ± 1.3, for ASD 17.3 ± 1.5, non-significant). For the body ROIs and for the non-social ROIs there were no significant group differences for the two fixation variables (see **Supplementary Table 6**).

## Discussion

We set out to investigate social perception in autism spectrum disorder groups under the framework of the current Bayesian autism spectrum disorder theories and using, to our knowledge for the first time in this field, dynamic gaze modeling with Bayesian Hidden Markov Models (BHMM) on real-life scenes. We found that gaze transitions between faces were significantly less in the ASD+ADHD group compared to TD. Interestingly, transitions between faces were also less likely, in terms of probabilities, to occur in the ASD+ADHD group. Regarding established gaze variables, fixation count to faces was reduced in ASD+ADHD, whereas visit count to faces did not differ across groups. Moreover, the ASD group showed similar social visual perception to TD in the studied variables. Finally, there were no differences between groups in the non-social transitions and fixation variables. Therefore, the hypothesis of reduced social perception in participants with autism spectrum disorder was confirmed particularly for the comorbid group and only for the face regions.

Transition variables, reflecting a more dynamical aspect of gaze, were sensitive to group differences and seem to reflect atypicalities of social visual perception. According to our results, the reduced amount and likelihood of social transitions did not come with an overall reduced visit count in the ROIs, indicating that differences found in transition variables are a consequence of reduced gaze linkages between the stimulus elements. Besides, it is important to note that the regression analysis controlled for total transition count. Our results confirm recent suggestions in the eye tracking literature, that not only the fixations per se, but also the dynamical linkage of fixation clusters contribute to individual differences in gazing behavior (21). Surprisingly, there is a gap in autism spectrum disorder literature exploring transitions between social elements in social scenes. A possible reason for this lack of studies investigating social transition may be the fact that the importance of presenting social interaction has been highlighted only recently (48). Therefore, the majority of studies until then have been focusing on a quantification of attention in isolated social elements of the presented stimuli. Thus, our study supports the measurement of social transitions as an objective variable for investigating social perception in autism spectrum disorder.

The present study has a special bearing for current influential theories of autism spectrum disorder (7). Specifically, the hypothesis of “Predictive Impairment in Autism” (6) is associated with Markov models and the hypothesized impaired capability of individuals with autism spectrum disorder to evaluate the probabilistic links within their environment. In our example, the social elements of each scene represent the hidden states of a Markov process and the probabilistic links between them are reflected by the respective transition probabilities. Hence, if it is more likely to visually link two faces that interact with each other and this behavior is considered typical for social understanding, then in case this linkage is less likely, the social understanding might be atypical. This atypical, or even impaired, understanding could presumably lead to more frequent “surprises” – or prediction errors –, an outcome of social interactions that is usually undesired (7). Thus, the approach introduced here composes an illustrative example of the appropriateness of a Bayesian framework on social visual perception in autism spectrum disorder research.

Here, we incorporate not only conceptual but also methodological advantages. Social perception is regarded and analyzed as a dynamical process that unfolds in space and time, thus being more representative of the evolving gaze trajectories (49). To deal with this complexity in the analysis of eye-tracking data, few dynamical approaches have been previously proposed. Based on the calculation of transition and entropy measures, children with autism spectrum disorder were shown to utilize immature exploration strategies for faces (50). Another study, applied networking analyses on transition data showing that autism spectrum disorder participants differ from controls in the aspect of centrality when exploring faces (51). Expanding on these viewpoints, our study shows following strengths. *Firstly*, participants were presented full-featured real-life social scenes and not isolated faces, following recommendations regarding the importance of naturalistic assessments for quantifying atypicalities in social attention in autism spectrum disorder (52). *Secondly*, with the adopted methodology, generation of ROIs additionally included the temporal dimension of each single fixation duration, enabling a more detailed analysis of gaze movements (27). *Thirdly*, the representative model of ROIs considered all participants’ individual gaze trajectories and thus accounts for the heterogeneity of the studied clinical populations and the importance of individual differences in scene viewing (53, 54). Hence, the demonstrated analysis extends the potentials of dynamical approaches in this field.

Furthermore, in accordance with recent studies suggesting that social impairments are more pronounced in comorbid cases compared to individuals without this comorbidity (55–57), here only the ASD+ADHD comorbid group manifested reduced social perception both in transition and fixation variables. However, it is important to consider that until the release of the new diagnostic guidelines (1), few eye tracking studies in social cognition distinguished between these groups (19, 58). Therefore, it is unclear if previous studies recruited participants with comorbid ASD+ADHD in their ASD groups and if the overall performance of the reported ASD group was influenced from the comorbid participants. This might also explain why some studies find similar performance of ASD and TD (17). Our results, regarding the ASD group agree with these studies suggesting a similar social perception to the TD group. Another explanation for these results might be the fact that our study included only participants with high-functioning autism spectrum disorder which reportedly have milder symptoms (59) and thus did not show differences in these variables with TD. On the contrary, it has been suggested that ASD+ADHD individuals have greater treatment demand (60). Therefore, we contribute to the understanding of social perception in the comorbid group which needs to be further investigated.

This study has several caveats. The implemented method has been utilized for studying healthy adults and has not been previously applied to clinical groups or children. It thus might ignore potential assumptions made during conceptualization. Further, the limited sample size of this exploratory analysis did not allow replication of the results. Concerning the clinical groups, potential factors influencing visual processing, such as IQ, education, age or visual processing deficits, were not considered in the statistical analysis, again due to the limited sample size. Moreover, the real-life scenes presented in this study explicitly did not include any extreme facial emotion expressions in order to avoid further bias of attentional gaze. Yet, difficulties in recognizing subtle or complex emotions have been reported for participants with autism spectrum disorder (61). Thus, a possible influence of emotion expression to the gaze behavior of our participants cannot be ruled out and therefore future studies could investigate effects of emotions in further detail and in real-life scenes. Furthermore, the presence of the experimenter in the cabin, although assuring engagement and safety of participants, should be also considered as a limitation of the study as it could have an impact on social anxiety and task performance. Particularly it is shown that fear or anxiety of negative evaluation have an effect on gaze (62) and therefore the impact of anxiety which is also a common comorbid condition in ASD should be also controlled for (63). Finally, this analysis distinguishes between social and non-social transitions and compares each of these categories between groups, while the transitions between social and non-social ROIs are not addressed and thus, the range of the gaze behavior is not fully covered.

Implications, for the design of future studies arise not only *via* the inclusion of measurements such as transition variables, but also from the entire analysis approach. The interpretable measurements presented in combination with a promising theoretical background, could for example aid in a step by step visual training of social perception, thus objectifying treatment outcomes and contributing to the development of risk assessments (48, 64). Given the flexibility of the social scene presentation paradigm and the practicality of eye tracking technique, such trainings could be developed specifically for children, customized for different developmental ages and levels of difficulties (65). This can eventually facilitate the realization of longitudinal studies and monitoring of long-time interventions (66, 67). Moreover, our method can be extended by combining other sensory inputs, neurophysiological measurements or other functions such as decision making (68–70). Finally, multimodal analysis of social interactions and real-life settings will be of great importance and could represent the everyday challenges of the autism spectrum disorder population in a better way (71, 72).

In conclusion, we show that Bayesian theories provide a reliable approach to study social perception deficits of autism spectrum disorder and we contribute to social cognition in autism spectrum disorder research in various aspects: *theoretically*, current Bayesian theories are here demonstrated in an interpretable example of social visual perception (to our knowledge for the first time); *methodologically*, an innovative Bayesian Modeling is applied, contributing to the further development of advanced dynamical gaze analysis; and *clinically*, practical measurements are presented with the scope of developing more accurate interventions for social skills in children and adults with autism spectrum disorder.

## Data Availability Statement

The stimuli supporting the conclusions of this article will be made available by the authors upon request, without undue reservation.

## Ethics Statement

The studies involving human participants were reviewed and approved by Ethik-Kommission der Albert-Ludwigs-Universität Freiburg, University Freiburg, Freiburg, Germany. Written informed consent to participate in this study was provided by the participants’ legal guardian/next of kin. Written informed consent was obtained from the individual(s) for the publication of any potentially identifiable images or data included in this article.

## Author Contributions

CI—data collection, conceptualization, data analysis, statistical analyses, original draft writing. DS—study design, data collection. M-ES—statistical analyses, manuscript writing. MB—study design, manuscript writing. LT—manuscript writing, review, and editing. CF—funding Acquisition, manuscript editing. GB—statistical analyses, data analysis, manuscript writing, review, and editing CK—study design, contributed data, manuscript editing. All authors contributed to the article and approved the submitted version.

## Funding

This research was supported by a grant of the Research Commission of the Medical Faculty of the University of Freiburg (KLE1076/16) and the State Funded Doctoral Scholarship of Baden-Wuerttemberg. The article processing charge was funded by the University of Freiburg in the funding programme Open Access Publishing.

## Conflict of Interest

The authors declare that the research was conducted in the absence of any commercial or financial relationships that could be construed as a potential conflict of interest.
