# Supplementary material for: Social Visual Perception Under the Eye of Bayesian Theories in Autism Spectrum Disorder Using Advanced Modeling of Spatial and Temporal Parameters
Source: Front Psychiatry. 2020 Sep 23;11:585149. doi: 10.3389/fpsyt.2020.585149 (PMC7546363; doi:10.3389/fpsyt.2020.585149)

Supplementary Material

# Supplementary Data

Details on diagnostic tests and screenings applied are described below.

- For the Autism Spectrum Disorder (ASD) diagnosis the Autism Diagnostic Observation Schedule (ADOS, (1)) and the parent-rated semi-structured Autism Diagnostic Interview-Revised (ADI-R, (2)) were used.
- Moreover, Attention-Deficit/Hyperactivity Disorder (ADHD) symptoms in ASD patients were assessed based on the International Statistical Classification of Diseases and Related Health Problems (ICD-10) and the DSM-IV adapted to the local native language, with the parent-rated external assessment questionnaire and the self-assessment questionnaire questionnaires (DISYPS-I), parent-rated external assessment questionnaire (EAQ) and the self-assessment questionnaire (SAQ) questionnaires (both in: (3)).
- Typically Developing (TD) participants were recruited through local schools, sport groups and from the departmental database.
- All participants were screened with the parent-rated Child Behaviour Checklist (CBCL;(4)) and the Social Responsiveness Scale (SRS; (5)) to exclude psychiatric symptoms in TD.

References:

1. Rühl D, Bölte S, Feineis-Matthews S, Poustka F. *Diagnostische Beobachtungsskala für Autistische Störungen (ADOS)*. (2004).

2. Bölte, S., Rühl, D., Schmötzer, G., & Poustka F. *Diagnostisches interview für autismus - revidiert. German version of the autism diagnostic interview - revised (ADI-R)*. (2005).

3. Döpfner, M., Görtz-Dorten, A., Lehmkuhl, G., Breuer, D., & Goletz H. *Diagnostik-System für psychische Störungen nach ICD-10 und DSM-IV für Kinder und Jugendliche*. Huber (2008).

4. Achenbach T, Rescorla L. *Manual for the ASEBA school-age forms & profiles: Child behavior checklist for ages 6-18, teacher’s report form, youth self-report*. (2001).

5. Bölte S, Poustka F, Constantino JN, Gruber CP. SRS: Skala zur Erfassung sozialer Reaktivität: dimensionale Autismus-Diagnostik. *Verlag Hans Huber* (2005)

# Supplementary Tables

## Supplementary Table 1.

Fixed effect tests of total transitions and total fixation duration used as covariates

| **A. Total transitions** | | |  | **B. Total fixation duration** | | |
| --- | --- | --- | --- | --- | --- | --- |
|  | **F** | **p-value** |  |  | **F** | **p-value** |
| group | 0.2146 | 0.808 |  | group | 1.918 | 0.158 |
| actors | 0.0395 | 0.843 |  | actors | 0.365 | 0.549 |
| group ✻ actors | 0.1848 | 0.832 |  | group ✻ actors | 0.940 | 0.398 |

## Supplementary Table 2.

Mean, standard deviation and range of social and non-social transition count

| A. Total social transition count | | | | | | B. Total non-social transition count | | | |
| --- | --- | --- | --- | --- | --- | --- | --- | --- | --- |
|  | **Mean** | **SD** | **Min** | **Max** |  | **Mean** | **SD** | **Min** | **Max** |
| TD | 96 | 26 | 45 | 143 |  | 146 | 41 | 80 | 250 |
| ASD | 96 | 21 | 63 | 129 |  | 144 | 40 | 100 | 210 |
| ASD+ADHD | 83 | 23 | 52 | 129 |  | 150 | 44 | 81 | 218 |
| Abbreviations: SD=Standard deviation, TD =typically developed, ASD = Autism Spectrum Disorder, ADHD= Attention-Deficit/Hyperactivity Disorder | | | | | | | | | |

## Supplementary Table 3.

Transition counts - Statistical results from mixed effect model

**A. Faces to faces**

| **Fixed Effect tests** | | | |  |  |  |
| --- | --- | --- | --- | --- | --- | --- |
|  | | **F** | | **p-value** |  |  |
| Group | | 4.86 | | 0.00788 |  |  |
| actors | | 53.35 | | 4E-13 |  |  |
| Total transitions | | 7.9 | | 0.00503 |  |  |
| Group ✻ actors | | 3.3 | | 0.03725 |  |  |
|  | |  | |  |  |  |
| **Post Hoc Tests - Group** | | | |  |  |  |
| **Group** | | | **Difference** | **SE** | **t** | **p-value** |
| ASD - ASD+ADHD | | | 2.657 | 1.25 | 2.131 | 0.0333 |
| TD - ASD | | | 0.557 | 1.13 | 0.493 | 0.6221 |
| TD - ASD+ADHD | | | 3.215 | 1.05 | 3.057 | 0.0023 |
|  | | |  |  |  |  |
| **Estimated Marginal Means** | | | |  | **95% Confidence Interval** | |
| **Group** | **Mean** | | | **SE** | **Lower** | **Upper** |
| TD | 8.38 | | | 0.644 | 7.12 | 9.64 |
| ASD | 7.82 | | | 0.929 | 6 | 9.64 |
| ASD+ADHD | 5.17 | | | 0.831 | 3.53 | 6.8 |

**B. Bodies to bodies**

| **Fixed Effect tests** | | | |  |  |  |
| --- | --- | --- | --- | --- | --- | --- |
|  | | **F** | | **p-value** |  |  |
| Group | | 0.624 | | 0.5367 |  |  |
| actors | | 67.788 | | 1.9E-15 |  |  |
| Total transitions | | 9.92 | | 0.0026 |  |  |
| Group ✻ actors | | 1.461 | | 0.23297 |  |  |
|  | |  | |  |  |  |
| **Post Hoc Tests - Group** | | | |  |  |  |
| **Group** | | | **Difference** | **SE** | **t** | **p-value** |
| ASD - ASD+ADHD | | | 1.162 | 1.76 | 0.659 | 0.5107 |
| TD - ASD | | | 0.496 | 1.6 | 0.31 | 0.7567 |
| TD - ASD+ADHD | | | 1.657 | 1.49 | 1.114 | 0.2665 |
|  | | |  |  |  |  |
| **Estimated Marginal Means** | | | |  | **95% Confidence Interval** | |
| **Group** | **Mean** | | | **SE** | **Lower** | **Upper** |
| TD | 10.2 | | | 0.91 | 8.4 | 12 |
| ASD | 9.7 | | | 1.313 | 7.11 | 12.3 |
| ASD+ADHD | 8.54 | | | 1.176 | 6.22 | 10.9 |

**C. Face to bodies**

| **Fixed Effect tests** | | | |  |  |  |
| --- | --- | --- | --- | --- | --- | --- |
|  | | **F** | | **p-value** |  |  |
| Group | | 3.1 | | 0.04604 |  |  |
| actors | | 174.14 | | 1E-16 |  |  |
| Total transitions | | 2.8 | | 0.10015 |  |  |
| Group ✻ actors | | 2.87 | | 0.05743 |  |  |
|  | |  | |  |  |  |
| **Post Hoc Tests - Group** | | | |  |  |  |
| **Group** | | | **Difference** | **SE** | **t** | **p-value** |
| ASD - ASD+ADHD | | | 0.814 | 0.345 | 2.364 | 0.0185 |
| TD - ASD | | | -0.26 | 0.312 | -0.831 | 0.4062 |
| TD - ASD+ADHD | | | 0.555 | 0.291 | 1.909 | 0.0568 |
|  | | |  |  |  |  |
| **Estimated Marginal Means** | | | |  | **95% Confidence Interval** | |
| **Group** | **Mean** | | | **SE** | **Lower** | **Upper** |
| TD | 2.44 | | | 0.178 | 2.09 | 2.79 |
| ASD | 2.7 | | | 0.257 | 2.2 | 3.2 |
| ASD+ADHD | 1.89 | | | 0.23 | 1.43 | 2.34 |

**D. Bodies to faces**

| **Fixed Effect tests** | | | |  |  |  |
| --- | --- | --- | --- | --- | --- | --- |
|  | | **F** | | **p-value** |  |  |
| Group | | 0.497 | | 0.60828 |  |  |
| actors | | 12.532 | | 0.00042 |  |  |
| Total transitions | | 5 | | 0.02562 |  |  |
| Group ✻ actors | | 0.522 | | 0.59378 |  |  |
|  | |  | |  |  |  |
| **Post Hoc Tests - Group** | | | |  |  |  |
| **Group** | | | **Difference** | **SE** | **t** | **p-value** |
| ASD - ASD+ADHD | | | 0.2744 | 0.339 | 0.8098 | 0.4184 |
| TD - ASD | | | -0.0109 | 0.307 | -0.0356 | 0.9716 |
| TD - ASD+ADHD | | | 0.2634 | 0.286 | 0.9219 | 0.357 |
|  | | |  |  |  |  |
| **Estimated Marginal Means** | | | |  | **95% Confidence Interval** | |
| **Group** | **Mean** | | | **SE** | **Lower** | **Upper** |
| TD | 1.218 | | | 0.175 | 0.875 | 1.56 |
| ASD | 1.229 | | | 0.252 | 0.734 | 1.72 |
| ASD+ADHD | 0.955 | | | 0.226 | 0.511 | 1.4 |

**E. Non-social to non-social**

| **Fixed Effect tests** | | | |  |  |  |
| --- | --- | --- | --- | --- | --- | --- |
|  | | **F** | | **p-value** |  |  |
| Group | | 0.3318 | | 0.71767 |  |  |
| actors | | 7.5333 | | 0.00609 |  |  |
| Total transitions | | 46.0155 | | 1E-11 |  |  |
| Group ✻ actors | | 0.0525 | | 0.94887 |  |  |
|  | |  | |  |  |  |
| **Post Hoc Tests - Group** | | | |  |  |  |
| **Group** | | | **Difference** | **SE** | **t** | **p-value** |
| ASD - ASD+ADHD | | | -0.465 | 0.593 | -0.785 | 0.4322 |
| TD - ASD | | | 0.166 | 0.536 | 0.309 | 0.7571 |
| TD - ASD+ADHD | | | -0.3 | 0.5 | -0.599 | 0.5492 |
|  | | |  |  |  |  |
| **Estimated Marginal Means** | | | |  | **95% Confidence Interval** | |
| **Group** | **Mean** | | | **SE** | **Lower** | **Upper** |
| TD | 4.79 | | | 0.306 | 4.19 | 5.39 |
| ASD | 4.63 | | | 0.441 | 3.76 | 5.49 |
| ASD+ADHD | 5.09 | | | 0.395 | 4.32 | 5.87 |

## Supplementary Table 4.

Transition probabilities - Statistical results from mixed effect model

**A. Faces to faces**

| **Fixed Effect tests** | | | | |  | |  | |  | |  |
| --- | --- | --- | --- | --- | --- | --- | --- | --- | --- | --- | --- |
|  | | **F** | | | **p-value** | |  | |  | |  |
| Group | | 3.214 | | | 0.0406685 | |  | |  | |  |
| actors | | 14.807 | | | 0.0001274 | |  | |  | |  |
| Group ✻ actors | | 0.993 | | | 0.37096301 | |  | |  | |  |
|  | |  | | |  | |  | |  | |  |
| **Post Hoc Tests - Group** | | | | |  | |  | |  | |  |
| **Group** | | | **Difference** | | **SE** | | **t** | | **p-value** | |  |
| ASD - ASD+ADHD | | | | 0.0411 | | 0.0372 | | 1.11 | | 0.269246408 | |
| TD - ASD | | | 0.0393 | | 0.0342 | | 1.15 | | 0.251771625 | |  |
| TD - ASD+ADHD | | | 0.0804 | | 0.0318 | | 2.52 | | 0.01185452 | |  |
|  | | |  | |  | |  | |  | |  |
| **Estimated Marginal Means** | | | | |  | | **95% Confidence Interval** | | | |  |
| **Group** | **Mean** | | | | **SE** | | **Lower** | | **Upper** | |  |
| TD | 0.301 | | | | 0.02 | | 0.262 | | 0.34 | |  |
| ASD | 0.262 | | | | 0.0277 | | 0.207 | | 0.316 | |  |
| ASD+ADHD | 0.221 | | | | 0.0248 | | 0.172 | | 0.269 | |  |

**B. Bodies to bodies**

| **Fixed Effect tests** | | | |  |  |  |
| --- | --- | --- | --- | --- | --- | --- |
|  | | **F** | | **p-value** |  |  |
| Group | | 0.375 | | 0.68782293 |  |  |
| actors | | 54.695 | | 7.596E-13 |  |  |
| Group ✻ actors | | 0.148 | | 0.86277345 |  |  |
|  | |  | |  |  |  |
| **Post Hoc Tests - Group** | | | |  |  |  |
| **Group** | | | **Difference** | **SE** | **t** | **p-value** |
| ASD - ASD+ADHD | | | 0.036 | 0.0428 | 0.842 | 0.400770805 |
| TD - ASD | | | -0.0139 | 0.0388 | -0.358 | 0.72069047 |
| TD - ASD+ADHD | | | 0.0221 | 0.0361 | 0.614 | 0.540069342 |
|  | | |  |  |  |  |
| **Estimated Marginal Means** | | | |  | **95% Confidence Interval** | |
| **Group** | **Mean** | | | **SE** | **Lower** | **Upper** |
| TD | 0.377 | | | 0.0221 | 0.333 | 0.42 |
| ASD | 0.391 | | | 0.0319 | 0.328 | 0.453 |
| ASD+ADHD | 0.355 | | | 0.0285 | 0.299 | 0.411 |

**C. Face to bodies**

| **Fixed Effect tests** | | | |  |  |  |
| --- | --- | --- | --- | --- | --- | --- |
|  | | **F** | | **p-value** |  |  |
| Group | | 1.4 | | 0.247038033 |  |  |
| actors | | 71.38 | | 8E-16 |  |  |
| Group ✻ actors | | 2.29 | | 0.103204597 |  |  |
|  | |  | |  |  |  |
| **Post Hoc Tests - Group** | | | |  |  |  |
| **Group** | | | **Difference** | **SE** | **t** | **p-value** |
| ASD - ASD+ADHD | | | -0.01846 | 0.0165 | -1.116 | 0.265858225 |
| TD - ASD | | | -0.0049 | 0.0142 | -0.345 | 0.730132498 |
| TD - ASD+ADHD | | | -0.02336 | 0.0141 | -1.663 | 0.097915589 |
|  | | |  |  |  |  |
| **Estimated Marginal Means** | | | |  | **95% Confidence Interval** | |
| **Group** | **Mean** | | | **SE** | **Lower** | **Upper** |
| TD | 0.11 | | | 0.00791 | 0.0947 | 0.126 |
| ASD | 0.115 | | | 0.01177 | 0.092 | 0.138 |
| ASD+ADHD | 0.134 | | | 0.01159 | 0.1108 | 0.156 |

**D. Bodies to faces**

| **Fixed Effect tests** | | | |  |  |  |
| --- | --- | --- | --- | --- | --- | --- |
|  | | **F** | | **p-value** |  |  |
| Group | | 0.1281 | | 0.87984926 |  |  |
| actors | | 0.028 | | 0.86718089 |  |  |
| Group ✻ actors | | 0.1579 | | 0.85401331 |  |  |
|  | |  | |  |  |  |
| **Post Hoc Tests - Group** | | | |  |  |  |
| **Group** | | | **Difference** | **SE** | **t** | **p-value** |
| ASD - ASD+ADHD | | | 0.00724 | 0.0227 | 0.32 | 0.74950293 |
| TD - ASD | | | 0.00223 | 0.0209 | 0.107 | 0.91498076 |
| TD - ASD+ADHD | | | 0.00948 | 0.019 | 0.499 | 0.61799227 |
|  | | |  |  |  |  |
| **Estimated Marginal Means** | | | |  | **95% Confidence Interval** | |
| **Group** | **Mean** | | | **SE** | **Lower** | **Upper** |
| TD | 0.0907 | | | 0.0119 | 0.0672 | 0.114 |
| ASD | 0.0884 | | | 0.0171 | 0.0547 | 0.122 |
| ASD+ADHD | 0.0812 | | | 0.0147 | 0.0521 | 0.11 |

**E. Non-social to non-social**

| **Fixed Effect tests** | | | |  |  |  |
| --- | --- | --- | --- | --- | --- | --- |
|  | | **F** | | **p-value** |  |  |
| Group | | 0.353 | | 0.70270688 |  |  |
| actors | | 42.907 | | 7.1514E-11 |  |  |
| Group ✻ actors | | 0.341 | | 0.71139753 |  |  |
|  | |  | |  |  |  |
| **Post Hoc Tests - Group** | | | |  |  |  |
| **Group** | | | **Difference** | **SE** | **t** | **p-value** |
| ASD - ASD+ADHD | | | -0.012 | 0.0165 | -0.7272 | 0.46906969 |
| TD - ASD | | | 0.0116 | 0.015 | 0.7773 | 0.43909319 |
| TD - ASD+ADHD | | | -3.44e−4 | 0.0139 | -0.0247 | 0.98031686 |
|  | | |  |  |  |  |
| **Estimated Marginal Means** | | | |  | **95% Confidence Interval** | |
| **Group** | **Mean** | | | **SE** | **Lower** | **Upper** |
| TD | 0.237 | | | 0.00855 | 0.22 | 0.254 |
| ASD | 0.225 | | | 0.01229 | 0.201 | 0.249 |
| ASD+ADHD | 0.237 | | | 0.01098 | 0.216 | 0.259 |

## Supplementary Table 5.

Fixation count- Statistical results from mixed effect model

**A. Fixations to faces**

| **Fixed Effect tests** | | | |  |  |  |
| --- | --- | --- | --- | --- | --- | --- |
|  | | | **F** | **p-value** |  |  |
| Group | | | 2.692 | 0.069 |  |  |
| actors | | | 1.839 | 0.176 |  |  |
| total fixation duration | | | 1.29 | 0.257 |  |  |
| Group ✻ actors | | | 0.363 | 0.696 |  |  |
|  | | |  |  |  |  |
| **Post Hoc Tests - Group** | | | |  |  |  |
| **Group** | | **Difference** | | **SE** | **t** | **p-value** |
| ASD - ASD+ADHD | | 6.75 | | 3.32 | 2.032 | 0.044 |
| TD - ASD | | -1.05 | | 3.03 | -0.347 | 0.729 |
| TD - ASD+ADHD | | 5.7 | | 2.83 | 2.018 | 0.046 |
|  | |  | |  |  |  |
| **Estimated Marginal Means** | | | |  | **95% Confidence Interval** | |
| **Group** | **Mean** | | | **SE** | **Lower** | **Upper** |
| TD | 27.7 | | | 1.73 | 24.3 | 31.1 |
| ASD | 28.7 | | | 2.48 | 23.8 | 33.6 |
| ASD+ADHD | 22.0 | | | 2.22 | 17.6 | 26.3 |

**B. Fixations to bodies**

| **Fixed Effect tests** | | | |  |  |  |
| --- | --- | --- | --- | --- | --- | --- |
|  | | | **F** | **p-value** |  |  |
| Group | | | 0.2349 | 0.791 |  |  |
| actors | | | 13.7836 | 0.0003 |  |  |
| total fixation duration | | | 0.0327 | 0.857 |  |  |
| Group ✻ actors | | | 1.4282 | 0.243 |  |  |
|  | | |  |  |  |  |
| **Post Hoc Tests - Group** | | | |  |  |  |
| **Group** | | **Difference** | | **SE** | **t** | **p-value** |
| ASD - ASD+ADHD | | 2.020 | | 4.40 | 0.459 | 0.648 |
| TD - ASD | | 0.509 | | 4.03 | 0.126 | 0.900 |
| TD - ASD+ADHD | | 2.528 | | 3.75 | 0.674 | 0.503 |
|  | |  | |  |  |  |
| **Estimated Marginal Means** | | | |  | **95% Confidence Interval** | |
| **Group** | **Mean** | | | **SE** | **Lower** | **Upper** |
| TD | 24.1 | | | 2.30 | 19.5 | 28.7 |
| ASD | 23.6 | | | 3.29 | 17.0 | 30.2 |
| ASD+ADHD | 21.6 | | | 2.94 | 15.7 | 27.5 |

**C. Fixations to non-social ROIs**

| **Fixed Effect tests** | | | |  |  |  |
| --- | --- | --- | --- | --- | --- | --- |
|  | | | **F** | **p-value** |  |  |
| Group | | | 0.0984 | 0.907 |  |  |
| actors | | | 2.8985 | 0.089 |  |  |
| total fixation duration | | | 0.9897 | 0.321 |  |  |
| Group ✻ actors | | | 0.0652 | 0.937 |  |  |
|  | | |  |  |  |  |
| **Post Hoc Tests - Group** | | | |  |  |  |
| **Group** | | **Difference** | | **SE** | **t** | **p-value** |
| ASD - ASD+ADHD | | -1.3119 | | 3.38 | -0.3878 | 0.700 |
| TD - ASD | | 1.2617 | | 3.11 | 0.4055 | 0.687 |
| TD - ASD+ADHD | | -0.0502 | | 2.90 | -0.0173 | 0.986 |
|  | |  | |  |  |  |
| **Estimated Marginal Means** | | | |  | **95% Confidence Interval** | |
| **Group** | **Mean** | | | **SE** | **Lower** | **Upper** |
| TD | 30.2 | | | 1.76 | 26.7 | 33.7 |
| ASD | 28.9 | | | 2.54 | 23.8 | 34.0 |
| ASD+ADHD | 30.2 | | | 2.27 | 25.7 | 34.8 |

## Supplementary Table 6.

Visit count - Statistical results from mixed effect model

**A. Visits to faces**

| **Fixed Effect tests** | | | |  |  |  |
| --- | --- | --- | --- | --- | --- | --- |
|  | | | **F** | **p-value** |  |  |
| Group | | | 0.755 | 0.473 |  |  |
| actors | | | 11.665 | 0.001 |  |  |
| total fixation duration | | | 0.229 | 0.633 |  |  |
| Group ✻ actors | | | 0.279 | 0.757 |  |  |
|  | | |  |  |  |  |
| **Post Hoc Tests - Group** | | | |  |  |  |
| **Group** | | **Difference** | | **SE** | **t** | **p-value** |
| ASD - ASD+ADHD | | 2.43 | | 1.99 | 1.223 | 0.224 |
| TD - ASD | | -1.18 | | 1.82 | -0.652 | 0.516 |
| TD - ASD+ADHD | | 1.25 | | 1.69 | 0.738 | 0.462 |
|  | |  | |  |  |  |
| **Estimated Marginal Means** | | | |  | **95% Confidence Interval** | |
| **Group** | **Mean** | | | **SE** | **Lower** | **Upper** |
| TD | 16.1 | | | 1.04 | 14.0 | 18.2 |
| ASD | 17.3 | | | 1.48 | 14.3 | 20.2 |
| ASD+ADHD | 14.8 | | | 1.33 | 12.2 | 17.5 |

**B. Visits to bodies**

| **Fixed Effect tests** | | | |  |  |  |
| --- | --- | --- | --- | --- | --- | --- |
|  | | | **F** | **p-value** |  |  |
| Group | | | 0.145 | 0.865 |  |  |
| actors | | | 17.048 | <0.001 |  |  |
| total fixation duration | | | 0.139 | 0.710 |  |  |
| Group ✻ actors | | | 1.186 | 0.308 |  |  |
|  | | |  |  |  |  |
| **Post Hoc Tests - Group** | | | |  |  |  |
| **Group** | | **Difference** | | **SE** | **t** | **p-value** |
| ASD - ASD+ADHD | | 0.6713 | | 1.71 | 0.3935 | 0.695 |
| TD - ASD | | 0.0830 | | 1.56 | 0.0532 | 0.958 |
| TD - ASD+ADHD | | 0.7542 | | 1.45 | 0.5189 | 0.606 |
|  | |  | |  |  |  |
| **Estimated Marginal Means** | | | |  | **95% Confidence Interval** | |
| **Group** | **Mean** | | | **SE** | **Lower** | **Upper** |
| TD | 10.8 | | | 0.890 | 9.07 | 12.6 |
| ASD | 10.8 | | | 1.274 | 8.22 | 13.3 |
| ASD+ADHD | 10.1 | | | 1.140 | 7.81 | 12.4 |

**C. Visits to non-social ROIs**

| **Fixed Effect tests** | | | |  |  |  |
| --- | --- | --- | --- | --- | --- | --- |
|  | | | **F** | **p-value** |  |  |
| Group | | | 0.293 | 0.747 |  |  |
| actors | | | 5.074 | 0.025 |  |  |
| total fixation duration | | | 1.063 | 0.304 |  |  |
| Group ✻ actors | | | 0.622 | 0.537 |  |  |
|  | | |  |  |  |  |
| **Post Hoc Tests - Group** | | | |  |  |  |
| **Group** | | **Difference** | | **SE** | **t** | **p-value** |
| ASD - ASD+ADHD | | 0.485 | | 1.001 | 0.484 | 0.63 |
| TD - ASD | | -0.702 | | 0.918 | -0.765 | 0.448 |
| TD - ASD+ADHD | | -0.217 | | 0.856 | -0.254 | 0.801 |
|  | |  | |  |  |  |
| **Estimated Marginal Means** | | | |  | **95% Confidence Interval** | |
| **Group** | **Mean** | | | **SE** | **Lower** | **Upper** |
| TD | 10.1 | | | 0.521 | 9.03 | 11.1 |
| ASD | 10.8 | | | 0.749 | 9.27 | 12.3 |
| ASD+ADHD | 10.3 | | | 0.671 | 8.94 | 11.6 |

# Supplementary Figures

**Supplementary Figure S1.**


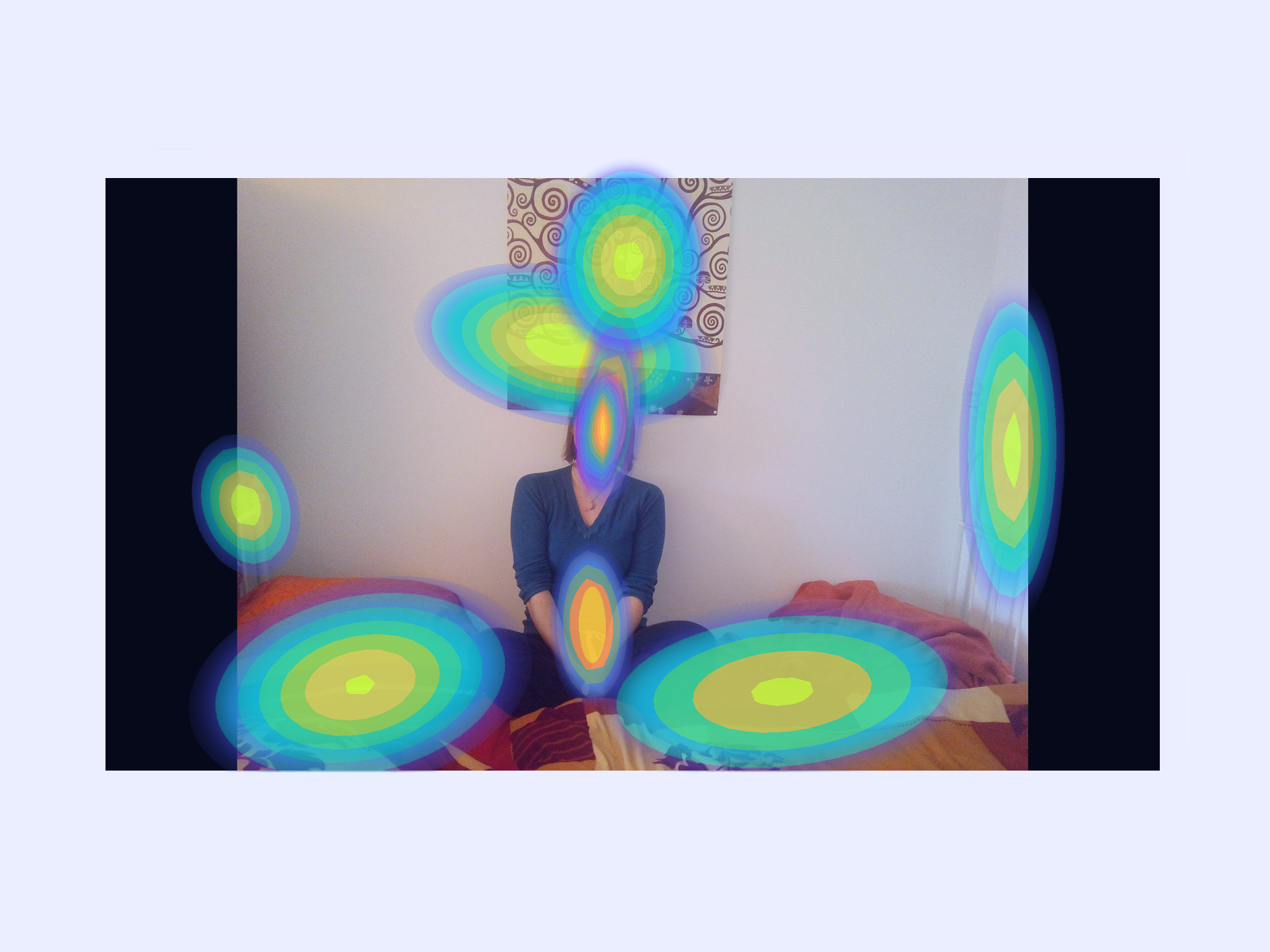


**Generated ROIs for stimulus B**. The ROIs of stimulus B formed from the gaze data of all participants are shown as Gaussian distributions, categorized in “social” (composed by the face and the body of the depicted person, purple tint) and “non-social” (remaining ROIs, cyan tint).

**Supplementary Figure S2.**

**Boxplot of transition probabilities for the typically developing (cyan), Autism Spectrum Disorder (yellow) and Autism Spectrum Disorder + Attention-Deficit/Hyperactivity Disorder (red) participants. The hiehgt of the barplots corresponds to the mean estimate from the mixed effects analysis, while the whiskers to the standard error. Significant difference was found for the face-to-face transition probabilities (See supplementary Table S4).**


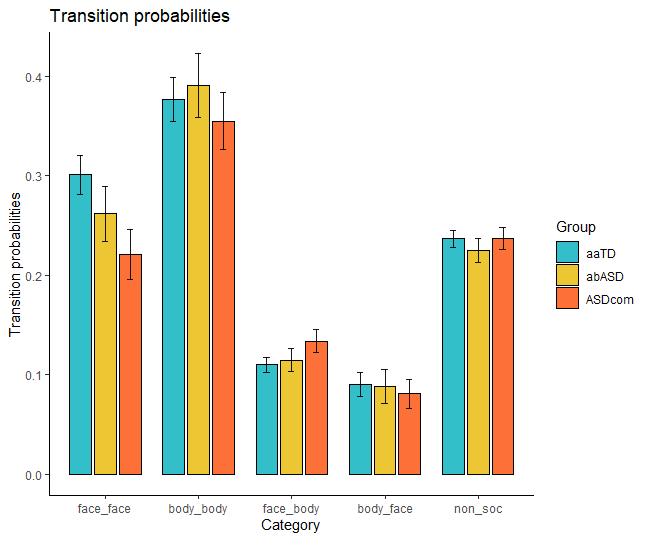

Supplement: Supplementary file 1 [file DataSheet_1.docx]
